# A Soft-Hard Combination-Based Cooperative Spectrum Sensing Scheme for Cognitive Radio Networks

**DOI:** 10.3390/s150204388

**Published:** 2015-02-13

**Authors:** Nhu Tri Do, Beongku An

**Affiliations:** 1 Department of Electronic & Computer Engineering in Graduate School, Hongik University, Sejong 339-701, Korea; E-Mail: dotrinhu@gmail.com; 2 Department of Computer & Information Communications Engineering, Hongik University, Sejong 339-701, Korea

**Keywords:** cognitive radio, spectrum sensing, soft combination, hard combination, likelihood ratio test, weighted decision

## Abstract

In this paper we propose a soft-hard combination scheme, called SHC scheme, for cooperative spectrum sensing in cognitive radio networks. The SHC scheme deploys a cluster based network in which Likelihood Ratio Test (LRT)-based soft combination is applied at each cluster, and weighted decision fusion rule-based hard combination is utilized at the fusion center. The novelties of the SHC scheme are as follows: the structure of the SHC scheme reduces the complexity of cooperative detection which is an inherent limitation of soft combination schemes. By using the LRT, we can detect primary signals in a low signal-to-noise ratio regime (around an average of −15 dB). In addition, the computational complexity of the LRT is reduced since we derive the closed-form expression of the probability density function of LRT value. The SHC scheme also takes into account the different effects of large scale fading on different users in the wide area network. The simulation results show that the SHC scheme not only provides the better sensing performance compared to the conventional hard combination schemes, but also reduces sensing overhead in terms of reporting time compared to the conventional soft combination scheme using the LRT.

## 1. Introduction

### 1.1. Motivation

In order to address the issue of spectrum scarcity that is encountered in the current frequency allocation policy of wireless communication systems, cognitive radio [1] has been considered as a promising means for improving efficient spectrum usage. Using cognitive radio (CR), the secondary users (SUs) are allowed to use the spectrum that is allocated to primary users (PUs) when the primary users are temporarily not using it. More specifically, according to IEEE 802.22 standard, customer premise equipment (CPE) Wireless Regional Area Network (WRAN) devices which are considered as the secondary users, will use the vacant channels in the VHF and UHF bands that are allocated to the Television Broadcasting Service in the frequency range between 54 MHz and 862 MHz while avoiding interference to the broadcast incumbents, which are considered as primary users, in these bands.

In order to prevent harmful interference to the primary users in a certain spectrum, the secondary users have to perform spectrum sensing before they start to access that spectrum. In addition, before starting transmitting in that spectrum, the SUs have to satisfy the predefined sensing results that are requirements of the PUs. Therefore, spectrum sensing plays a key role in cognitive radio technology. Local sensing methods for individual SUs have been studied, and generally based on any of these techniques: energy detection [2], matched filtering [3], and cyclostationary feature detection [4]. Each of such methods has different requirements and advantages and disadvantages. Cyclostationary detection requires knowledge of the cyclic frequency of the primary signal while matched filtering requires the information of waveforms and channels of primary users. If such information is not available, energy detection can be applied since the primary signals are assumed to be random.

In cognitive radio, SUs have to be able to detect very weak signals from the primary users. This is difficult for individual spectrum sensing since the fundamental characteristics of wireless channels such as multipath fading, shadowing, can degrade the received signal. Specifically, accurate detection is impossible below a certain SNR level which is known as the SNR wall [5]. Cooperative spectrum sensing is proposed to overcome these issues of local spectrum sensing. In centralized cooperative detection, SUs send their local sensing information to the fusion center (FC) where the final decision on existence of a primary signal is made. According to the type of information that SUs provide to the FC, cooperative spectrum sensing schemes can be generally categorized into two kinds: soft combination schemes and hard combination schemes [6].

In hard combination scheme, SUs first turn the local decisions into one-bit decision, *i.e.*, 0 or 1 implies that a primary user is absent or present, respectively, based on their observations of the primary signal. Then, they send these one-bit decisions to the fusion center. Using hard combination in cooperative detection not only reduces the communication cost, but also is easy to implement. However, using soft combination can have the cooperative sensing performance improvement over hard combination [7]. In soft combination scheme, SUs directly send their local observations which are energy values of the received signals from the primary user to the fusion center.

Recently, the Likelihood Ratio Test (LRT)-based soft combination scheme for cooperative spectrum sensing has attracted considerable attention [8–12]. In [8], the authors proposed a linear test based on the LRT detector, and investigated the proposed test under several primary signal and channel statistics scenarios. The analysis in [9] was focused on a maximum eigenvalue-based Likelihood Ratio Test under the cases of known and unknown noise levels of primary signal. In [10], the authors studied a distributed Likelihood Ratio Test detector for spectrum sensing while the channels are treated as random channels with a Nakagami-Lognormal mixture distribution. Then, they further investigated the cases of frequency selective Nakagami channels in [11], where the correlation of frequency domain gains is taken into account. In [12], the authors proposed the optimal LRT for detecting digitally modulated signals of primary users based on Bayesian rules. However, the arguments made against the use of a soft combination scheme are that the bandwidth requirement for reporting channels scales gradually with the size of the network [13].The disadvantaged aspects of soft combination schemes have also been discussed in our previous work [14].To minimize the bandwidth of the control channel, certain local processing is required [15]. Therefore, hard combination schemes should be considered in which only one-bit local decisions are forwarded to the common center by SUs. However, some studies have proved that soft combination yields more precise detection than hard combination [6].

Different from the other related works, in this paper we propose a soft-hard combination scheme which makes use of both soft combination scheme and hard combination scheme together. In [16], a hard combination scheme using a weighted decision fusion rule not only provides good sensing performance, but also reduces the sensing time. Due to cost and bandwidth considerations, the hard decision combination is an attractive option that should be utilized. Therefore, we consider a cluster-based cognitive radio network in which LRT-based soft combination scheme is applied in each cluster. Specifically, the cluster head of each cluster combines sensing observations from other SU members and makes the cluster decision by using the LRT. In order to reduce sharing bandwidth, only cluster heads send the one-bit cluster decisions to the fusion center. The use of the LRT needs the SNR of primary user at the SU which conducts this test. This average SNR is assumed to be known since the transmission loss between two nodes can be obtained by using location awareness [13,17]. Location information has been applied in hard combination scheme for cooperative detection [13] or in concurrence transmission in cognitive radio networks [18].Since we consider the large network where each cluster experiments a different primary signal SNR, the weighted decision fusion rule is used at the fusion center for distinguishing the different contribution of each cluster to the global decision at the fusion center.

### 1.2. Contributions

In this paper, we propose a soft-hard combination scheme, called SHC scheme, for cooperating spectrum sensing schemes in cognitive ratio networks. The following are the main contributions of the study presented in our paper:

- The SHC scheme based on Likelihood Ratio Test (LRT) utilizes both soft combination and hard combination schemes. In each cluster, the LRT can provide better sensing performance compared to conventional soft combination scheme using an Energy Detector. In the whole network, the SHC scheme achieves better sensing performance compared to conventional hard combination schemes using the *k-out-of-N* fusion rule or the LRT at the fusion center. In addition, the SHC scheme can reduce the reporting time of sensing data compared to the conventional soft combination scheme using the LRT.
- We not only minimize the false alarm probability, but also maximize the detector probability of cluster heads by utilizing the Minimum Error Probability (MEP) criterion to obtain the optimal cluster threshold. In most of related works, e.g., [8,10,11], LRT is based on the Neyman-Pearson theorem which maximizes only the detection probability for a given false alarm probability. The optimal threshold of cluster head in our paper is derived numerically.
- The use of soft combination provides enough statistics for cluster head to conduct a LRT while the use of hard combination reduces the cost and bandwidth for cooperative sensing process.

To the best of our knowledge, the LRT based soft-hard combination scheme has not been available in previous related works.

## 2. The Proposed Soft-Hard Combination Scheme: SHC Scheme

In this section, we present system model of the proposed soft-hard combination (SHC) scheme. Two stages of spectrum sensing processing, *i.e.*, soft combination at each cluster and hard combination at the fusion center, are mathematically described.

### 2.1. System Description

**Figure 1.**
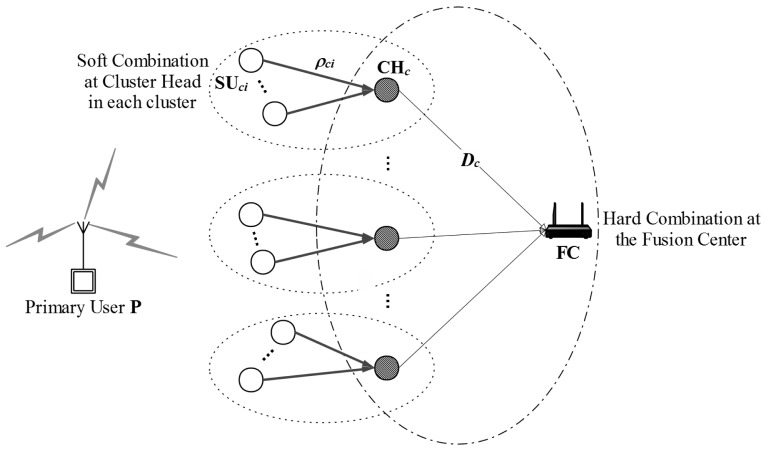
The soft-hard combination (SHC) scheme in which P represents the primary user, SU*_ci_* represents the *i*-th SU in the *c*-th cluster, ρ*_ci_* represents its local test statistic which is the received energy contents of the primary signal, CH*_c_* represents the *c*-th cluster head, *D_c_* represents its one bit cluster decision, and FC represents the fusion center.

Let us consider a cognitive radio network consisting of *K* clusters in which each cluster has the same number of SUs, denoted by *N*. There is a Fusion Center (FC) that organizes the clusters, chooses cluster heads, and collaborates all SUs in the network. The secondary system works under the radio range of a primary user P. The primary user may be present or absent, but its status does not change during a single sensing interval. We assume that all the SUs in each cluster have the same average SNR of the received primary signal. This assumption is reasonable since clusters are built by grouping the adjacent SUs that are located in a same small area. However, each cluster experiments difference channel condition of the link between itself and primary user P. Thus, each cluster has independent and difference average SNR of the primary signal. Cooperative spectrum sensing process of SHC scheme consists of two stages which are described in Figure 1.

In the first stage, cluster heads make a cluster decision on the primary activity by using a soft combination as follows: at the beginning of the sensing process, the *i*-th SU in the *c*-th cluster SU*_ci_* listens to the primary signal, and makes its local test statistic ρ*_ci_* which is the energy content of the received signal. We assume that each SU will utilize *M* primary signal samples for making the local test statistics. Then, the local test statistic ρ*_ci_* is sent to a cluster head. We assume that each cluster has one cluster head that is capable for collaborating with all remaining SUs in that cluster. Denote CH*_c_*, *c* = 1, 2, …, *K*, as the cluster head of the *c*-th cluster. We suggest the cluster head selection as follows: in order to be aware of the presence of PU, the CR system performs spectrum sensing periodically. Generally, the frame structure of CR system consists of one sensing slot and one data transmission slot. The cooperative spectrum sensing process is carried out periodically by the FC in the sensing slot. The frequency of the cooperative spectrum sensing process depends on the system designer's consideration on application requirements, trade-offs between spectrum sensing and spectrum sharing, *etc.* Without the loss of generality, the FC randomly chooses a certain SU in each cluster as a cluster head for the corresponding clusters. It is reasonable since all SUs in the same cluster have the equal role because we assume that they have identical average SNRs of the received primary signal.

Next, the cluster heads conduct the Likelihood Ratio Test (LRT) based on the test statistics of all SUs in cluster including its own one and make the cluster decision on the existence of the PU into one bit hard decision. Let *D_c_*, *c* = 1, 2, …, *K*, denotes the cluster decision of the *c*-th cluster, *i.e.*, *D_c_* = 1 or *D_c_* = 0 refers to primary user is present or absent, respectively.

In the second stage, all cluster heads send their cluster decisions *D_c_* to the fusion center on error-free reporting channels. The fusion center then combines all the cluster decisions and makes the global decision by using the weighted decision fusion rule. As we mentioned before, since clusters experience difference average SNRs of the received primary signal, their contributions to the global decision will be also different. However, the conventional fusion rule *k-out-of-N* [19], e.g., OR rule, AND rule or MAJORITY rule do not consider this aspect. Therefore, *k-out-of-N* rule cannot be applied for the SHC scheme. On the other hand, the weighted decision fusion rule allocates different weighted factors to corresponding cluster decisions according to their sensing reliabilities.

**Figure 2.**
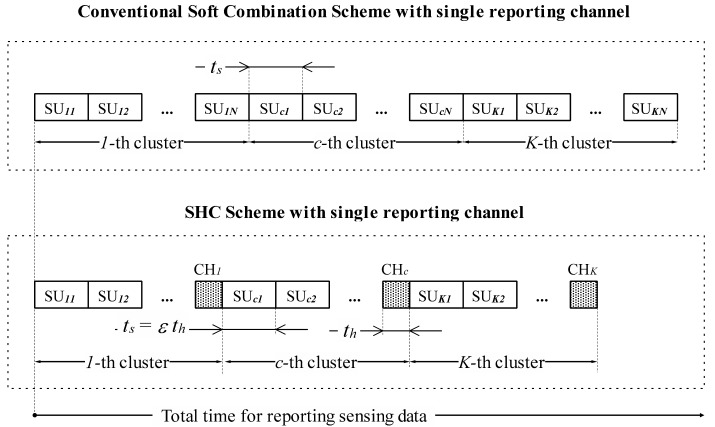
Reporting mechanism of SHC scheme, in which *t_s_* denotes the time for sending a test statistic, *t_h_* denotes the time for sending a one bit decision, ε is correlation coefficient between *t_s_* and *t_h_*.

The reporting mechanism of SHC scheme is depicted in Figure 2. In a conventional soft combination scheme, SUs sequentially send their sensing data to the FC. Let *t_s_* denote the transmission time that a single SU needs to forward its sensing data to the fusion center. On the other hand, in the SHC scheme, SUs in a same cluster send their sensing data to a cluster head. For fair comparison, the time that a SU forwards its sensing data to a cluster head is assumed also as *t_s_*. Then cluster heads make cluster decisions into one bit and sequentially send them to the FC. Let *t_h_* denote the transmission time that a CH needs to send its decision to the FC. For a given bandwidth and transmission rate of a control channel, the more data a SU reports to a cluster head, the more transmission time it needs. Therefore, let ε (ε > 0) be the correlation coefficient between the transmission time of unquantized information (soft sensing data) collected by a SU and the transmission time of one bit decision made by a CH, *i.e.*, *t_s_* = ε*t_h_*.

Finally, the global decision is made by the fusion center. Let *D_g_* denote the global decision for each sensing period, *i.e.*, *D_g_* = 1 or *D_g_* = 0 refers to primary user is present or absent, respectively. At the end of spectrum sensing process, FC broadcasts the global decision to the all the SUs in network. For the whole paper, Pr(A) denotes the probability of an arbitrary event A. For notational convenience, we use LRT to represent the Likelihood Ratio Test and L-LRT to represent the Log-Likelihood Ratio Test throughout this paper.

### 2.2. Soft Combination at Cluster Head in Each Cluster

The *i*-th secondary user of *c*-th cluster SU*_ci_* observes a received signal *r_ci_* over a sensing interval of *M* samples. We denote the signal transmitted by the primary user by *s_ci_*. This signal is propagated to SU*_ci_* over a flat fading channel that is time invariant over *M* sampling intervals. The *m*-th sample of the discrete received signal *r_ci_*(*m*) at the secondary user SU*_ci_* can be represented as:

(1) $$r_{ci}\left(m\right)=\left\{ \begin{array}{ll} n_{ci}\left(m\right), & H_{0}\\ h_{ci}s_{ci}\left(m\right)+n_{ci}\left(m\right), & H_{1} \end{array}\right.$$

where *H*_0_ is the hypothesis that the PU is absent and *H*_1_ is the hypothesis that the PU is present in the vicinity of the SUs. *r_ci_* is the primary received signal at the *i*-th SU in the *c*-th cluster SU*_ci_*. The noise is assumed to be additive, white and Gaussian (AWGN) with zero-mean and known variance $\sigma_{n,ci}^{2}$, *i.e.*, $n_{ci}\left(m\right)\sim N\left(0,\sigma_{n,ci}^{2}\right)$, and *h_ci_* represents the channel gain which is assumed to be constant during the detection interval, *s_ci_* is the transmitted primary signal. We assume that *s_ci_* and *n_ci_* are independent, which is reasonable from a practical perspective. Additionally, we assume that the status of primary user is unchanged during a single sensing interval as in those literatures [8,10,12].

The local test statistic which is estimation of received primary signal power of the SU*_ci_* can be written as:

(2) $$\rho_{ci}=\sum_{m=1}^{M}{\left|r_{ci}\left(m\right)\right|}^{2}$$

where *M* = 2*TW* is the number of collected samples at each SU in one sensing interval in which *T* and *W* correspond to detection time and signal bandwidth in Hertz, respectively. In the proposed scheme, only one channel is sensed at one time.

The test statistics of SUs are then combined at the corresponding cluster head by using Equal Gain Combining (EGC). The cluster test statistic which is also known as the estimation of received primary signal power at the cluster head SC*_c_* of the *c*-th cluster is given as:

(3) $$\rho_{c}=\sum_{i=1}^{N}\sum_{m=1}^{M}{\left|r_{ci}\left(m\right)\right|}^{2}$$

Under the hypothesis *H*_0_, the test statistic ρ*_c_* is an independent random variable whose probability density function (*pdf*) is a Chi-square distribution with *L* degrees of freedom, where *L* = *NM*. Under hypothesis *H*_1_, ρ*_c_* is the independent non-central chi-square random variable with *L* degrees of freedom and non-central parameter γ*_c_L*.

The average SNR of primary user's signal measured at cluster head CH*_c_* is represented as:

(4) $$\gamma_{c}=y_{c1}=\cdots =\gamma_{cN}=\frac{1}{M}\sum_{m=1}^{M}\frac{{\left|h_{ci}\right|}^{2}{\left|s_{ci}\left(m\right)\right|}^{2}}{\sigma_{n,ci}^{2}},i=1,2,\ldots ,N$$

Note that we assume that all SUs in the same cluster have identical SNR.

As we mentioned before, the SNR is obtained by using the SU location information. For the ease of analysis, we assume that the noise has unit variance. By using the Central Limit Theorem (CLT), the distributions of the test statistic ρ*_c_* can be approximated by the Gaussian distributions under either *H_0_* or *H_1_*. Therefore, the distributions of ρ*_c_* are given as [20]:

(5) $$\rho_{c}\sim \left\{ \begin{array}{l} \begin{array}{cc} N\left(L,2L\right), & H_{0} \end{array}\\ \begin{array}{cc} N\left(L\left(1+\gamma_{c}\right),2L{\left(1+\gamma_{c}\right)}^{2}\right), & H_{1} \end{array} \end{array}\right.$$

Note that *L* = *NM* is the number of samples of the received primary signal. These samples are collected using soft combination at the cluster head. The cluster head of each cluster then uses ρ*_c_* as cluster observation to make cluster decision. The cluster head conducts the LRT to make the cluster decision on the absence or present of primary user. The log-likelihood ratio value for the binary hypothesis test given in Equation (1) can be represented as [5]:

(6) $$\Lambda_{c}=\log\frac{f_{\rho }\left(\rho_{c}|H_{1}\right)}{f_{\rho }\left(\rho_{c}|H_{0}\right)}$$

where *f*_ρ_(ρ*_c_*|*H*_1_) and *f*_ρ_(ρ*_c_*|*H*_0_) are the probability density functions of the cluster test statistic ρ*_c_* under hypothesis *H*_1_ and *H*_0_, respectively, and log refers to the natural logarithm. Since the SNRs of the received primary signals in a cluster are identical, the value Λ*_c_* of the test that is conducted at a certain secondary user *i*-th in *c*-th cluster SU*_ci_* and also at a cluster head CH*_c_* can be considered to be derived from the same distribution *f*_Λ_(Λ*_c_*). Hence, the random choice of cluster head is reasonable. Then, the cluster decision *D_c_* ∈ {0,1} is made based on the Log-Likelihood Ratio Test (L-LRT) as follows:

(7) $$\Lambda_{c}\begin{array}{c} \overset{H_{1}}{>}\\ \underset{H_{0}}{<} \end{array}\lambda_{c}$$

where λ*_c_* is the cluster threshold. The derivation of the cluster threshold is explained in detail in Section 3.2.

### 2.3. Hard Combination at the Fusion Center

Let us recall that we consider the network consisting of *K* clusters in which each cluster has *N* SUs. The fusion center receives and combines cluster decisions in order to determine the status of primary user. Here, the weighted decision fusion rule is adopted at the fusion rule. Specifically, the fusion rule adds a weighted factor *ω*_1_*_c_* into the cluster decision that refers to PU is present and a weighted factor ω_0_*_c_* into the cluster decision that refers to PU is absent before summing up all the weighted decisions. Denote *D* = [*D_1_*, *D_2_*, …, *D_K_*] as a set of received cluster decisions at the fusion center.

The fusion center makes the global decision by using the LRT as [21]:

(8) $$\frac{\mathrm{Pr}\left(D_{1},D_{2},\ldots ,D_{K}|H_{1}\right)}{\mathrm{Pr}\left(D_{1},D_{2},\ldots ,D_{K}|H_{0}\right)}=\frac{\mathrm{Pr}\left(D|H_{1}\right)}{\mathrm{Pr}\left(D|H_{0}\right)}\begin{array}{c} \overset{H_{1}}{>}\\ \underset{H_{0}}{<} \end{array}\frac{P_{0}}{P_{1}}$$

where *P*_0_ = Pr(*H*_0_) and *P*_1_ = Pr(*H*_1_) are the prior probabilities of the presence and absence of the PU signal, respectively, which are assumed to be known. We assume that the cluster heads' decisions are independent, after some algebra, the L-LRT is further expressed as:

(9) $$\sum_{c=1}^{K}\;\left[\left(1-D_{c}\right)\log\left(\frac{1-P_{d,c}}{1-P_{f,c}}\right)+D_{c}\log\left(\frac{P_{d,c}}{P_{f,c}}\right)\right]\begin{array}{c} \overset{H_{1}}{>}\\ \underset{H_{0}}{<} \end{array}\log\frac{P_{0}}{P_{1}}$$

Therefore, the weighted decision fusion rule can be rewritten in the form as follows:

(10) $$\sum_{c=1}^{K}\left[\left(1-D_{c}\right)\omega_{0c}+D_{c}\omega_{1c}\right]\begin{array}{c} \overset{H_{1}}{>}\\ \underset{H_{0}}{<} \end{array}\log\frac{P_{0}}{P_{1}}$$

where:

(11) $$\omega_{c}=\{\begin{array}{cc} \omega_{0c}=\log\frac{1-P_{d,c}}{1-P_{f,c}} & ifD_{c}=0\\ \omega_{1c}=\log\frac{P_{d,c}}{P_{f,c}} & ifD_{c}=1 \end{array}$$

Here, the weighted factors are selected by using Equation (11); the method is also presented in [13,21], which is based on the theorem proposed in [22]. Additionally, Equation (10) corresponds to the optimal decision fusion rule in [22]. Finally, the fusion center broadcasts the global decision which is resulted from Equation (10) to all the SUs in the network.

## 3. Optimal Cluster Threshold

In this section, we briefly introduce the Energy Detector (ED) which is the most common sensing method. In the rest of paper, we consider the Energy Detector as a conventional sensing method. Next, we provide the way to obtain the optimal cluster threshold for cluster heads in SHC scheme.

### 3.1. Energy Detector

In order to illustrate the operation of the conventional sensing method Energy Detector, we consider that Energy Detector is employed at a certain cluster head CH*_c_*. In that case, CH*_c_* make the decision based on an energy threshold λ*_ED,c_* as follows:

(12) $$D_{ED,c}=\{\begin{array}{cc} 1, & if\rho_{c}>\lambda_{ED,c}\\ 0, & if\rho_{c}<\lambda_{ED,c} \end{array}$$

where ρ*_c_* is test statistic which is formulated in (3). Herein, *D_ED,c_* = 1 or *D_ED,c_* = 0 mean that the hypotheses of *H*_1_ or *H*_0_ are decided at CH*_c_* by using the Energy Detector, respectively. The local false alarm probability $P_{f,c}^{ED}$ and the local detection probability $P_{d,c}^{ED}$ can be determined as:

(13) $$P_{f,c}^{ED}=\mathrm{Pr}\left(\rho_{c}>\lambda_{ED,c}|H_{0}\right)=Q\left(\frac{\lambda_{ED,c}-L}{\sqrt{2L}}\right)$$

and:

(14) $$P_{d,c}^{ED}=\mathrm{Pr}\left(\rho_{c}>\lambda_{ED,c}|H_{1}\right)=Q\left(\frac{\lambda_{ED,c}-L\left(1+\gamma_{c}\right)}{\sqrt{2L{\left(1+\gamma_{c}\right)}^{2}}}\right)$$

where γ*_c_* is the average SNR at CH*_c_*. Let us remind that *L* is the number of received primary samples that are collected at each CH*_c_*. $Q\left(x\right)=\int_{x}^{\infty }\left(1/\sqrt{2\pi }\right)\exp\left(t^{2}/2\right)dt$ is the *Q*-function.

### 3.2. Optimal Cluster Threshold

In order to compute the optimal cluster threshold, we need to derive the *pdf* of LRT value Λ*_c_*. In [23], a method has been presented to compute the *pdf* of the LRT value in general. This method will be used in our paper to determine the *pdf* of Λ*_c_*.

Let ρ = [ρ*_1_*, ρ*_2_*, …, ρ*_c_*, …, ρ*_K_*]. Let μ*_c,j_* and $\sigma_{c,j}^{2}$, *j* = 0 or *j* = 1, be means and variances of Equation (5). Note that ρ*_c_* is the random variable that represents the test statistic for LRT at the cluster head CH*_c_*. From Equations (5) and (6), the LRT value can be given as:

(15) $$\Lambda_{c}=\log\frac{\sigma_{c,0}}{\sigma_{c,0}}+\left(\frac{{\left(\rho_{c}-\mu_{c,0}\right)}^{2}}{2\sigma_{c,0}^{2}}-\frac{{\left(\rho_{c}-\mu_{c,1}\right)}^{2}}{2\sigma_{c,1}^{2}}\right)$$

Substituting the means and variances in Equation (5) into Equation (15), by applying the fundamental theorem [24], and after some algebra, the *pdf* of the LRT value can be derived as:

(16) $$f_{\Lambda }\left(\Lambda_{c}\right)=f_{\Lambda }\left(\Lambda_{c}|H_{0}\right)+f_{\Lambda }\left(\Lambda_{c}|H_{1}\right)$$

where:

(17) $$f_{\Lambda }\left(\Lambda_{c}|H_{0}\right)=\frac{P_{0}}{2\sqrt{\pi L\Delta }}\exp\left(-{\left(\frac{b+\sqrt{\Delta }}{2a}-L\right)}^{2}/4L\right)$$

and:

(18) $$f_{\Lambda }\left(\Lambda_{c}|H_{1}\right)=\frac{P_{1}}{2\left(\gamma_{c}+1\right)\sqrt{\pi L\Delta }}\exp\left(-\frac{1}{4L{\left(\gamma_{c}+1\right)}^{2}}{\left(\frac{b+\sqrt{\Delta }}{2a}-L\left(\gamma_{c}+1\right)\right)}^{2}\right)$$

when Λ*_c_* ≥ −*b*^2^/4*a* − *d*, otherwise, *f*_Λ_(Λ*_c_*|*H*_0_) = *f*_Λ_(Λ*_c_*|*H*_1_) = 0, where:

(19) $$\begin{array}{llll} a=\frac{\gamma_{c}\left(\gamma_{c}+2\right)}{4L\left(\gamma_{c}+1\right)}, & b=\frac{\gamma_{c}}{2\left(\gamma_{c}+1\right)}, & d=\log\left(\gamma_{c}+1\right), & \Delta =b^{2}+4a\left(d+\Lambda_{c}\right) \end{array}$$

The false alarm probability *P_f,c_* and the detection probability *P_d,c_* of each cluster head CH*_c_* are given as:

(20) $$P_{f,c}=\mathrm{Pr}\left(\Lambda_{c}>\lambda_{c}|H_{0}\right)=\int_{\lambda_{c}}^{\infty }f_{\Lambda }\left(\Lambda_{c}|H_{0}\right)d\Lambda$$

and:

(21) $$P_{d,c}=\mathrm{Pr}\left(\Lambda_{c}>\lambda_{c}|H_{1}\right)=\int_{\lambda_{c}}^{\infty }f_{\Lambda }\left(\Lambda_{c}|H_{1}\right)d\Lambda$$

The value of Equations (20) and (21) can be easily obtained by using MATLAB^®^ software of The MathWorks, Inc. (Natick, MA, USA). From the above discussions, we can see that the false alarm probability and detection probability of each cluster are determined by the channel condition, *i.e.*, the average SNR, and the cluster threshold. Given the fixed channel condition, it is meaningful to find an optimal local sensing threshold minimizing the global sensing error.

In this paper, we adopt the minimum error probability criterion [25–27] to determine the cluster threshold of *c*-th cluster which is given as:

(22) $$\lambda_{\text{opt},c}=\underset{\lambda_{c}}{\arg}\min\left(P_{0}P_{f,c}+P_{1}\left(1-P_{d,c}\right)\right)$$

As we can see in Equations (20)–(22), the optimal cluster threshold λ*_opt,c_* can be obtained based on the *pdf* of the LRT value Λ*_c_*. Therefore, by using the *pdf* in Equation (16) the cluster head CH*_c_* can obtain the optimal cluster threshold λ*_opt,c_* and then use it for the comparison in Equation (7).

## 4. Performance Evaluation

In this section, we first present sensing performance in terms of error probability *P_e,c_* of the soft combination using the Likelihood Ratio Test (LRT) at the cluster head CH*_c_*. We then compare the performances of soft combination using LRT to the one using the Energy Detector (ED) as a conventional sensing method at the same CH*_c_*. Next, we present the sensing performance of the hard combination at the fusion center in terms of total error probability *P_e_*. Finally, we provide the comparisons of sensing performance and reporting time between the SHC scheme, the conventional soft combination scheme, and the conventional hard combination schemes.

Our simulation is based on the Monte-Carlo method with 10^5^ iterations of the primary users' status. In order to simplify the performance analysis, we assume that clusters have the same number of nodes. It is an assumption adopted in the literature for cluster-based networks such as in [10,16]. In our simulation, we assume that there are five clusters in the network in which each cluster contains least two SUs. In each iteration, the probability of present and absence of PU signal is 0.5, *i.e.*, *P_1_* = *P_0_* = 0.5. The test statistic ρ*_c_* is modeled by using its *pdf* which is based on the number of primary received signal samples *M* = 50 samples at each local SU*_ci_* in a sensing interval, and its average SNR γ*_c_*. In our simulation, we consider the network that is divided into *K* = 5 clusters and we vary the value of number of SUs in one cluster *N*. The error probability *P_e,c_* at the CH*_c_* which is the summation of miss-detection probability, *P_m,c_* = 1 − *P_d,c_*, and false alarm probability *P_f,c_*, is as follows:

(23) $$P_{e,c}=P_{1}\left(1-P_{d,c}\right)+P_{0}P_{f,c}$$

Similarly, the total error probability *P_e_* at the fusion center (FC) is given as *P_e_* = *P_1_*(*1* − *P_d_* ) + *P_0_P_f_*, where *P_d_* and *P_f_* refer to the global detection probability and false alarm probability which are determined by simulation, respectively.

**Figure 3.**
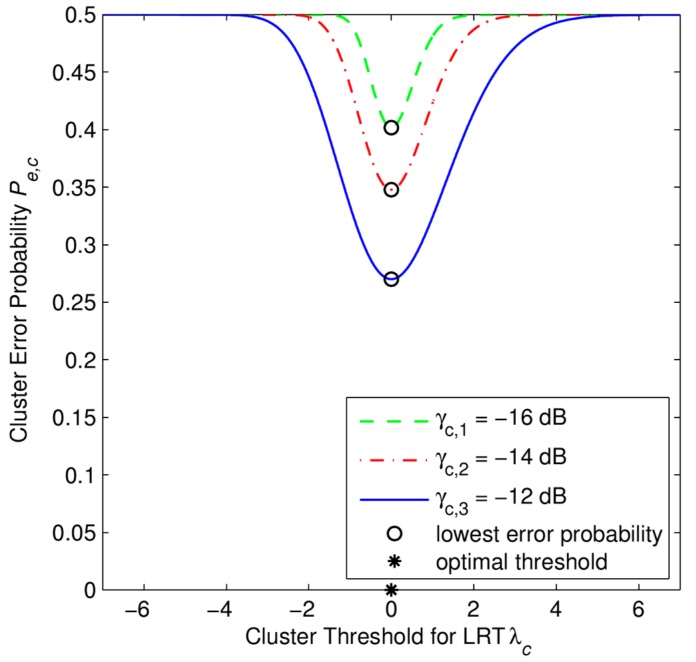
Derivation of the optimal cluster threshold in the SHC scheme. The error probability at a cluster head *P_e,c_* as a function of cluster threshold λ*_c_* with *P*_1_ = *P*_0_ = 0.5, *M* = 50 samples, *N* = 4 SUs.

**Figure 4.**
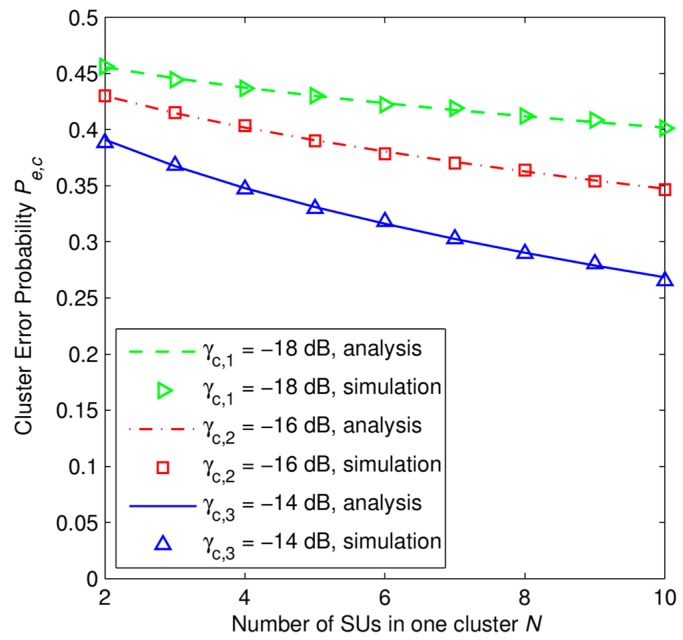
Error probability at a cluster head *P_e,c_* as a function of number of SUs in one cluster *N* with *P*_1_ = *P*_0_ = 0.5, *M* = 50 samples.

**Figure 5.**
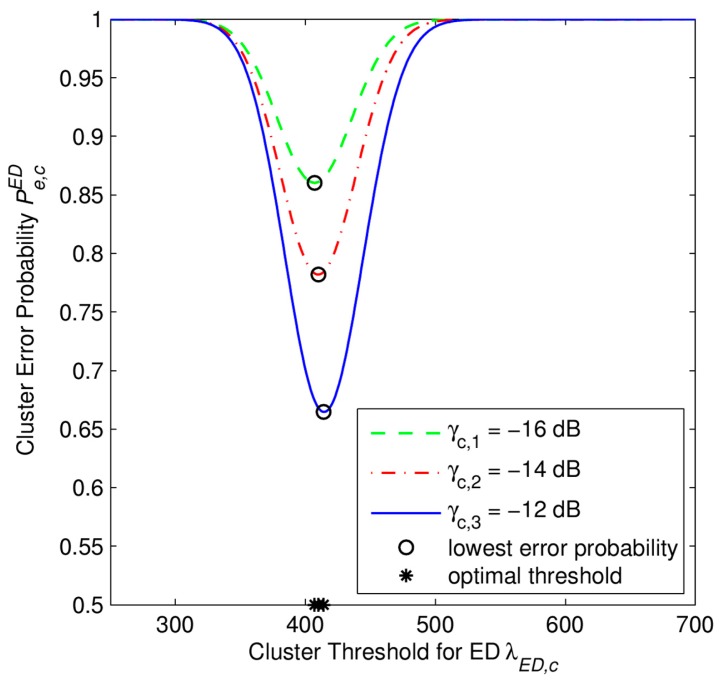
Derivation of the optimal cluster threshold in the case of using the Energy Detector (ED). The error probability at a cluster head $P_{e,c}^{ED}$ as a function of threshold λ*_ED,c_* with *P*_1_ = *P*_0_ = 0.5, *M* = 50 samples, *N* = 4 SUs.

**Figure 6.**
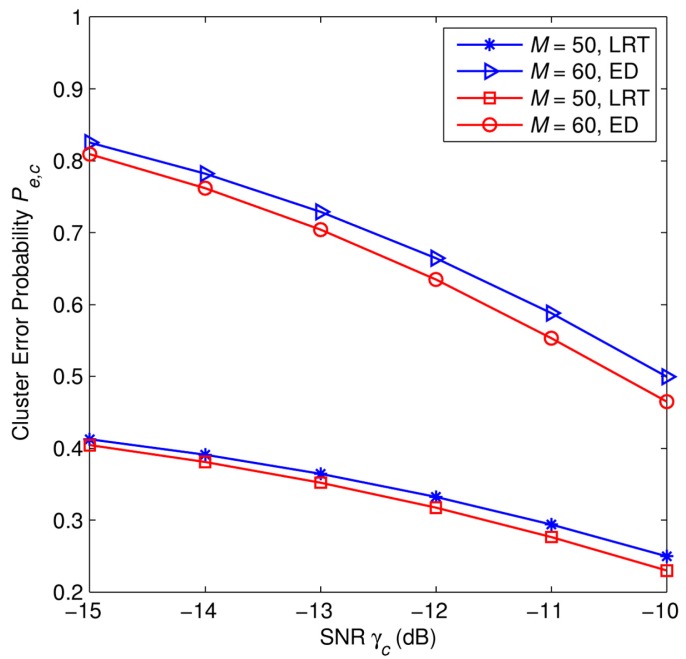
The comparison of sensing performances between soft combination scheme (at a cluster head) using the Likelihood Radio Test (LRT) and the one using the Energy Detector (ED) under different SNRs with *N* = 4 SUs.

From Figures 3, 4, 5 and 6, we investigate the performance of the soft combination in a certain cluster. In Figure 3, we depict how to obtain the optimal cluster threshold λ*_opt,c_* of the CH*_c_* in the certain *c*-th cluster. The optimal threshold λ*_opt,c_* is obtained by numerical calculation of *P_e,c_*. Simulation parameters are set as *N* = 4 SUs, *M* = 50 samples, and *γ_c,s_* refers to the average SNR (in dB) at *c*-th cluster under *s*-th scenario.

In Figure 4, we plot the error probability *P_e,c_* against with the number of SUs in one cluster with *M* = 50 samples, and under several scenarios of the average SNR γ*_c_*. Figure 4 shows that for a given value of the number of SUs in one cluster *N*, the error probability *P_e,c_* decreases along with the increase of the average SNR γ*_c_*. In addition, in any cases of the average SNR, the error probability *P_e,c_* curve goes down along with the increase of the number of SUs in one cluster *N*. It can be observed that the simulation results match well with the analytical results.

In Figures 3 and 4, we plot the error probability *P_e,c_* of a single cluster head under different received SNR levels. These simulation results can be considered as local results, i.e., there is no cooperation between clusters in the whole network, of the proposed scheme. The cause that leads to such a high error probability, *i.e.*, around 0.3, is the small number of cooperative secondary users, *i.e.*, SUs in only one cluster participate, and the small number of the received signal samples *M* from primary user that we set for each SU in the simulation, *i.e.*, *M* = 50 samples.

Next, we compare the performances of the soft combination using the Likelihood Ratio Test (LRT) to the one using the Energy Detector (ED) at the same CH*_c_*. Figure 5 depicts how to obtain the optimal local threshold λ*_opt,ED,c_* of ED at the certain CH*_c_* with *M* = 50 samples at each SU for several cases of the average SNRs. As we can see in Figure 5, the error probability of the SU using ED is really high since we consider the system under the low SNR regime. The results in Figure 5 are used to obtain the results in Figure 6.

Figure 6 plots the error probability as a function of SNR at CH*_c_*. In both the cases of ED and LRT, for a given value of SNR, the higher the number of samples *L* of primary signal, the lower the error probability we can achieve. The error probability decreases along with the increase of the SNR. In any case of SNR, the use of LRT significantly improves the sensing performance of cooperative detection compared to the use of ED, especially, in the low SNR regime. The gap between LRT and ED diminishes gradually as the SNR increases.

**Figure 7.**
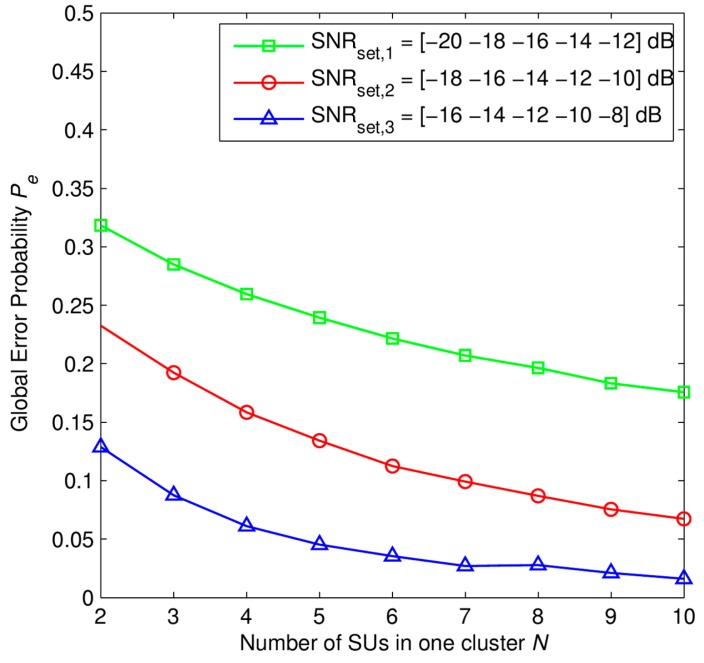
Global error probability *P_e_* as a function of number of SUs in one cluster *N* with *P*_1_ = *P*_0_ = 0.5, *M* = 50 samples, *K* = 5 clusters, SNR*_set_*_,_*_1_* = [−20 −18 −16 −14 −12] dB, SNR*_set_*_,_*_2_* = [−18 −16 −14 −12 −10] dB, SNR*_set_*_,_*_3_* = [−16 −14 −12 −10 −8] dB.

**Figure 8.**
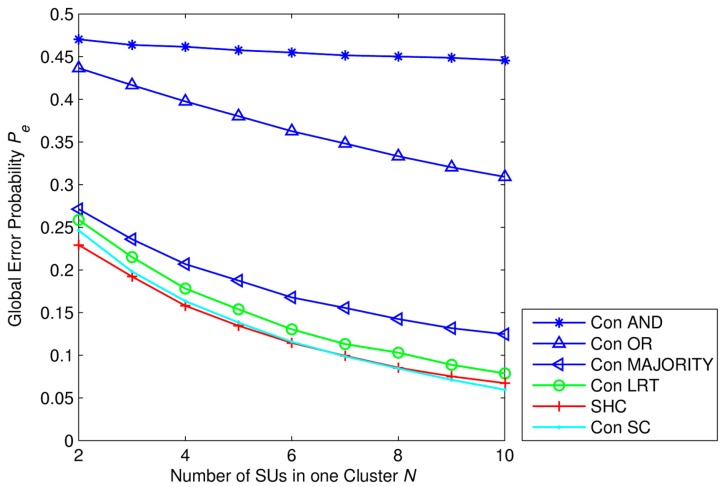
Global error probability *P_e_* as a function of number of SUs in one cluster, with *P*_1_ = *P*_0_ = 0.5, *M* = 50 samples, *K* = 5 clusters, SNR*_set_* = [−18 −16 −14 −12 −10] dB where Con AND, Con OR, Con MAJORITY, and Con LRT represent for the Conventional hard combination schemes using AND rule, OR rule, MAJORITY rule, and the LRT, respectively. Con SC represents for Conventional Soft Combination scheme using the LRT, and SHC represents for the proposed scheme.

In Figures 7 and 8, we investigate the performance of the SHC scheme. The global error probability *P_e_* at the fusion center is presented in Figure 7 as a function of number of SUs in one cluster. We consider the SHC scheme consisting of *K* = 5 clusters in which each cluster has *N* SUs. The different average SNRs of corresponding clusters are described in the SNR set that is denoted by SNR*_set_*. Each SNR set represents different scenarios of network environment in the SHC scheme. Here, three sets of the average SNR are presented as follows: SNR*_set_*_,_*_1_* = [−20 −18 −16 −14 −12] dB, SNR*_set,2_* = [−18 −16 −14 −12 −10] dB, and SNR*_set_*_,_*_3_* = [−16 −14 −12 −10 −8] dB. The number of received primary signal samples at each SU is *M* = 50 samples.

We can see that the global error probability increases when the channel conditions of the whole network is worse. However, global error probability is decreased when the network size is lager, *i.e.*, the number of SUs in each cluster is higher. It shows the novelty of SHC scheme when it applied for large area network with huge number of SUs.

Now, we compare performances of SHC scheme with hard combination scheme using *k-out-of-N* rule or the LRT and conventional soft combination scheme using the LRT. We define the conventional hard combination scheme as follows: all SUs make local decisions on the existence of PU into one bit hard by using ED and then send these one-bit decisions to the fusion center (FC). The *k-out-of-N* rule or the LRT are applied at the fusion center to make a global decision. By using the *k-out-of-N* rule, the fusion center decides the PU signal being transmitted, *i.e.*, *H*_1_, when there exists at least *n* out of *K* SUs inferring *H*_1_. Otherwise, the FC decides the PU signal not being transmitted, *i.e.*, *H*_0_. It can be seen that the OR rule, AND rule, and MAJORITY rule correspond to the case of *n* = 1, *n* = *K*, and *n* ≥ *K*/2, respectively. Let us note that here *k* and *N* are used as proper names of the fusion rule, and they are different from *k* and *N* that are used in our paper. We define the conventional soft combination scheme as follows: all SUs send their test statistics to the fusion center; the sensing data are then combined by using Equal Gain Combining (EGC); and the FC decides the existence of PU by using the LRT which is described in Equations (6) and (7).

Simulation parameters are set as *K* = 5 clusters with different average SNR values and *N* SUs in each cluster. SNR values of clusters are described in the set SNR*_set_* = [−18 −16 −14 −12 −10] dB. The number of received primary signal samples at each SU is *M* = 50 samples. From Figure 8, we can see that in any cases of network size, the global error probability of the SHC is the lower than the conventional hard combination schemes using AND rule, OR rule, MAJORITY rule, or the LRT. The performance gaps between the SHC scheme and other conventional hard combination schemes increases gradually as the network size increases. However, performances of the SHC scheme and the conventional soft combination scheme using LRT are not significantly different. Specifically, when the number of SUs in one cluster is small, *i.e.*, *N* < 4, the proposed scheme has a little bit better performance than the conventional one. But, when the number of SUs in one cluster increases, *i.e.*, *N* > 6, the SHC scheme is slightly worse than the conventional one. Note that the number of cluster in our simulation is fixed at *K* = 5 clusters.

**Figure 9.**
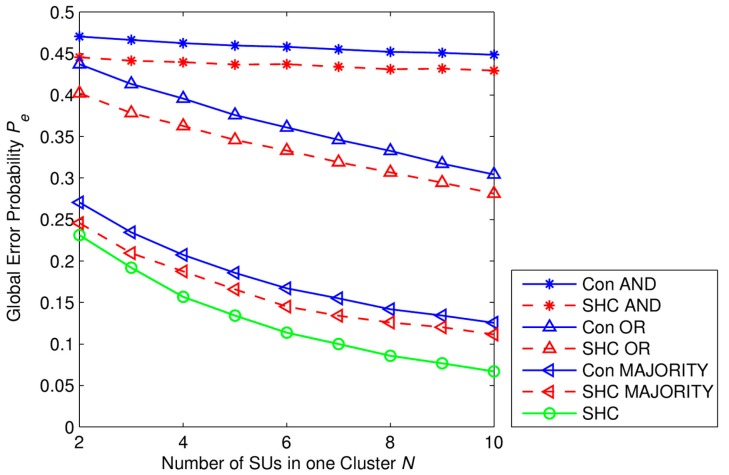
Global error probability *P_e_* as a function of number of SUs in one cluster, with *P*_1_ = *P*_0_ = 0.5, *M* = 50 samples, *K* = 5 clusters, SNR*_set_* = [−18 −16 −14 −12 −10] dB where Con AND, Con OR, Con MAJORITY represent for the Conventional hard combination scheme using AND rule, OR rule, MAJORITY rule respectively. SHC AND, SHC OR, SHC MAJORITY represent for the modified SHC scheme using AND rule, OR rule, MAJORITY rule at the fusion center, respectively. SHC represents for the proposed SHC scheme using the weighted decision fusion rule at the fusion center.

Figure 9 provides the comparison of the performances of three schemes as follows. The conventional hard combination scheme using *k-out-of-N* rule described above, the modified SHC scheme has same structure as the proposed scheme in which, however, the *k-out-of-N* rule is applied at the fusion center instead of the weighted decision fusion rule, and the proposed SHC scheme. As we can see in Figure 9, the proposed SHC scheme gives the lowest global error probability. The modified SHC scheme outperforms the conventional hard communication scheme since the LRT is better than the conventional ED. However, the gap between two these scheme decreases along with the increase of the number of the secondary users since the performance of the *k-out-of-N* rule depends on the total number of hard decisions at the fusion center. Note that in the modified SHC scheme, only cluster heads send their hard decisions to the fusion center.

From the results in Figures 7 and 9, we can see that, under different average SNRs from primary users, each cluster has a different sensing reliability that leads to a different contribution to the global decision at the fusion center. Besides that, both the cluster error probability *P_e,c_* and the global error probability *P_e_* are decreased when the number of SUs in one cluster is higher, respectively. Therefore, we can see that with the same PUs' signal samples collected at each SU, the higher of the number of SUs in one cluster is, the more PU's signal samples the cluster head has and so the better sensing performance is. That is one of the strong points of our proposed SHC scheme.

Finally, we compare the reporting time of our proposed scheme with the conventional hard combination schemes and the conventional soft combination scheme. In this case, we assume that the conventional soft combination is applied at the fusion center. The reporting mechanism of the conventional soft combination is depicted in Figure 2. In our paper, we assume that there is only one reporting channel for SUs exchange sensing information. Therefore, total time for reporting sensing data in convention soft combination scheme is given as *T_SC_* = *KNt_s_*. The time that a CH needs to receive sensing data from other SUs corresponds to the summation time that SUs cost for sending their sensing data to the cluster head. Here, *t_s_* denotes the time that individual SU needs to send its sensing data to a cluster head. Therefore, the total time that a CH needs to receive sensing data from other SUs is (*N* − 1)*t_s_*, where *N* is the number of SUs in a cluster. The time that CHs need to compute cluster decision is assumed to be negligible. The time that a CH needs to send its result to the FC is denoted by *t_h_*, therefore the total time that CHs report cluster decisions to the FC is *Kt_h_*, where *K* is the number of CHs in network. Total time for reporting sensing data in convention hard combination scheme is given as *T_HC_* = *KNt_h_*. And, total time for reporting sensing data in SHC scheme is given as *T_SHC_* = *K*(*N* − 1)*t_s_* + *Kt_h_*. The reporting time comparison (RTC) is given as:

(24) $$\begin{array}{cc} {\text{RTC}}_{\text{SHC}-\text{SC}}=\frac{T_{\text{SHC}}}{T_{\text{SC}}}100\left(\%\right), & {\text{RTC}}_{\text{HC}-\text{SHC}}=\frac{T_{\text{HC}}}{T_{\text{SHC}}}100\left(\%\right) \end{array}$$

where *RTC_SHC-SC_* is the time efficiency of the SHC scheme compared to the conventional soft combination scheme using LRT, and *RTC_HC-SHC_* is the time efficiency of the conventional hard combination schemes compared to the SHC scheme.

**Figure 10.**
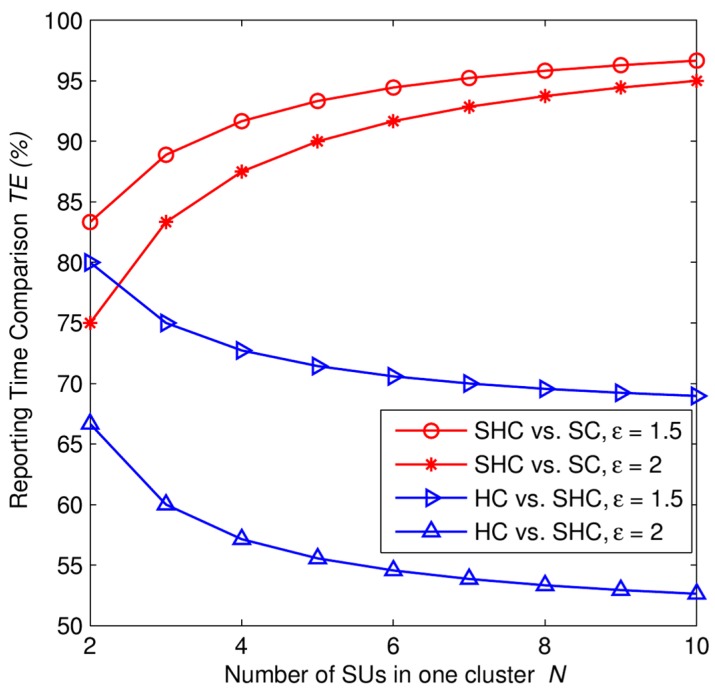
Time efficiency *TE* (%) as a function of the number of SUs in one cluster with *K* = 5 clusters

In Figure 10, we present the reporting time comparison as a function of SU in one cluster *N*. For a given bandwidth and transmission rate of a control channel, the more data a SU reports to a cluster head, the more transmission time it needs. Therefore, let ε (ε > 0) be the correlation coefficient between the transmission time of unquantized information (soft sensing data) collected by a SU and the transmission time of one bit decision made by a CH, i.e., t_s_ = εt_h_. It is obviously that *ε* increases when *M* increases.

In the comparison between the SHC scheme and the conventional soft combination scheme using LRT. As we can see in Figure 10, in any cases of network size and the value of *ε*, we always obtain the positive time efficiency. Specifically, the SHC scheme consumes only 93% of the needed time for the conventional soft combination scheme for a given value of *N* = 7 SUs, ε = 2. And, the time efficiency decrease as the network size increases. On the other hand, the bigger local test statistic is, *i.e.*, ε increases, the higher time efficiency we obtain in the SHC scheme.

On the contrary, in the comparison between the conventional hard combination schemes and the SHC scheme, we can observe that the SHC scheme need more 30% time for reporting data compared to the conventional one for a given value of *N* = 7 SUs and ε = 1.5. This additional time needed for the SHC scheme quickly increases to 46% compared to the time that the conventional one needs when ε = 2. Additionally, the more number of nodes in one cluster, the higher reporting time that the SHC needs compared to the conventional hard combination schemes. We can see that the amount of bit of sensing data strongly impact on the reporting time. It also shows that the use of hard combination consumes less cost than soft combination in terms of reporting time.

## 5. Conclusions

In this paper, we propose a Soft-Hard Combination (SHC) scheme that combines soft combination and hard combination into one spectrum sensing scheme. In the each cluster, cluster heads combine the test statistics of other SUs and conduct the Likelihood Ratio Test (LRT) with the optimal cluster threshold which is determined by the minimum error probability criterion. The optimal cluster threshold is derived by using the closed-form expression of the *pdf* of the LRT value. In addition, simulation results show that the LRT has better performance, especially in the low SNR regime, compared to the conventional Energy Detector (ED). Since different clusters experiment different SNRs of the received primary signal, they will have different contributions to the global decision. Therefore, by using the weighted decision fusion rule, the fusion center can distinguish the corresponding contributions of different cluster heads and the SHC scheme can achieve the better sensing performance compared to the conventional Hard Combination (HC) schemes with AND rule, OR rule, MAJORITY rule, and the LRT, respectively. However, the SHC scheme needs a bigger amount of reporting time than the conventional HC schemes. On contrary, the reporting mechanism of SHC scheme can reduce the reporting time compared to the conventional soft combination scheme using LRT. But sensing performances of these two schemes are not significantly different. Thus, we can see that there is a tradeoff between sensing performance in terms of error probability and sensing overhead in terms of reporting time.
